# Retraction Note: Sorcin Predicts Poor Prognosis and Promotes Metastasis by Facilitating Epithelial-mesenchymal Transition in Hepatocellular Carcinoma

**DOI:** 10.1038/s41598-018-29892-8

**Published:** 2018-08-02

**Authors:** Xiong Lei, Yahang Liang, Jian Chen, Shuai Xiao, Jian Lei, Jianfeng Li, Jinzhong Duanmu, Qunguang Jiang, Dongning Liu, Cheng Tang, Taiyuan Li

**Affiliations:** 1 Department of General Surgery, The First Affiliated Hospital of Nanchang University, Nanchang, 330006 China; 2 Department of Surgery, The First Affiliated Hospital of Sun Yat-Sen University, Guangzhou, 510080 China; 3 Liver Cancer Laboratory, Department of Surgery, Xiangya Hospital, Central South University, Changsha, 410008 Hunan China; 4 Department of Pathology, Affiliated Cancer Hospital of Xiangya School of Medicine, Central South University, Changsha, 410013 Hunan China

Retraction of: *Scientific Reports* 10.1038/s41598-017-10365-3, published online 30 August 2017

This Article is being retracted at the request of the corresponding author, because the authors did not obtain relevant permissions from the Liver Cancer Laboratory at Xiangya Hospital, Central South University, who is the owner of some of the clinical specimens and related data for the use of these materials in the study.

Xiong Lei, Shuai Xiao, and Taiyuan Li agreed to the retraction. The other authors could not be reached.

